# Immunosuppressive potential of human amnion epithelial cells in the treatment of experimental autoimmune encephalomyelitis

**DOI:** 10.1186/s12974-015-0322-8

**Published:** 2015-06-03

**Authors:** Courtney A. McDonald, Natalie L. Payne, Guizhi Sun, Leon Moussa, Christopher Siatskas, Rebecca Lim, Euan M. Wallace, Graham Jenkin, Claude C.A. Bernard

**Affiliations:** Australian Regenerative Medicine Institute, Monash University, Clayton, 3800 Australia; The Ritchie Centre, MIMR-PHI Institute of Medical Research, Clayton, VIC 3800 Australia; Monash Immunology and Stem Cell Laboratories, Monash University, Clayton, VIC 3800 Australia; Department of Anatomy and Developmental Biology, Monash University, Clayton, VIC 3800 Australia; Department of Obstetrics and Gynaecology, Monash University, Clayton, VIC 3800 Australia

**Keywords:** Amnion epithelial cells, Multiple sclerosis, Immunoregulation, Neurodegeneration, Demyelination, Stem cells

## Abstract

**Background:**

Multiple sclerosis (MS) is an autoimmune inflammatory disease of the central nervous system (CNS). In recent years, it has been found that cells such as human amnion epithelial cells (hAECs) have the ability to modulate immune responses in vitro and in vivo and can differentiate into multiple cell lineages. Accordingly, we investigated the immunoregulatory effects of hAECs as a potential therapy in an MS-like disease, EAE (experimental autoimmune encephalomyelitis), in mice.

**Methods:**

Using flow cytometry, the phenotypic profile of hAECs from different donors was assessed. The immunomodulatory properties of hAECs were examined in vitro using antigen-specific and one-way mixed lymphocyte proliferation assays. The therapeutic efficacy of hAECs was examined using a relapsing-remitting model of EAE in NOD/Lt mice. T cell responsiveness, cytokine secretion, T regulatory, and T helper cell phenotype were determined in the peripheral lymphoid organs and CNS of these animals.

**Results:**

In vitro, hAECs suppressed both specific and non-specific T cell proliferation, decreased pro-inflammatory cytokine production, and inhibited the activation of stimulated T cells. Furthermore, T cells retained their naïve phenotype when co-cultured with hAECs. In vivo studies revealed that hAECs not only suppressed the development of EAE but also prevented disease relapse in these mice. T cell responses and production of the pro-inflammatory cytokine interleukin (IL)-17A were reduced in hAEC-treated mice, and this was coupled with a significant increase in the number of peripheral T regulatory cells and naïve CD4+ T cells. Furthermore, increased proportions of Th2 cells in the peripheral lymphoid organs and within the CNS were observed.

**Conclusion:**

The therapeutic effect of hAECs is in part mediated by inducing an anti-inflammatory response within the CNS, demonstrating that hAECs hold promise for the treatment of autoimmune diseases like MS.

## Background

Multiple sclerosis (MS) is an inflammatory disease of the central nervous system (CNS) [1]. Current knowledge suggests that the disease is maintained by auto-reactive T cells that target proteins expressed predominantly in myelin and, to a lesser extent on axons, which ultimately results in CNS tissue injury [2]. A number of therapeutic approaches using immunomodulatory or immunosuppressive drugs such as interferon-β, glatiramer acetate, natalizumab, and Fingolimod (FTY720) have been designed to target the immune component of the disease process [3]. While these treatments are beneficial in halting the disease in approximately 30 % of relapsing-remitting (RR)-MS patients, they are only partially effective and have little impact on disease progression [4]. For this reason, there is a desperate need for alternative therapies to improve the outcomes for the majority of MS patients. Improved therapeutic outcomes will require the suppression of the inflammatory response, restoration of immunological tolerance, and the incorporation of neuroprotective strategies. For these reasons, stem cell therapy has gained momentum over the past decade as a potential treatment for MS.

One proposed stem cell source is human amnion epithelial cells (hAECs). These cells are isolated from the epithelial layer of the amniotic membrane, the innermost layer of the fetal membranes that surround the fetus [5]. The amnion is originally derived from embryonic ectoderm [6, 7] with differentiation of hAECs from the epiblast occurring around day 8 of human pregnancy, before gastrulation, at a time when the cells are still pluripotent. As a result of this early divergence, hAECs retain a high level of pluripotency as evidenced by the expression of several embryonic stem cell (ESC) markers including OCT-4, nanog, SSEA-3, SSEA-4, TRA 1-60, and c-kit [8–11]. hAECs are claimed to be immune privileged in so far as they do not express human leukocyte antigen (HLA) class II or co-stimulatory molecules [12, 13], theoretically making them potential candidates in allogeneic settings. Given that, on average, about 100–200 million hAECs can be isolated from a term placenta [13], these cells present an abundant source of potential regenerative tissue. Moreover, their collection does not hold ethical constraints in comparison with other stem cell sources such as ESCs. In vitro studies have shown that hAECs can generate clinically relevant cell types from ectoderm, mesoderm, and endoderm, such as cardiomyocytes, myocytes, osteocytes, adipocytes, pancreatic cells, hepatocytes, as well as neural and astrocytic cells [9, 10, 14]. More poignantly, investigations into their immunomodulatory properties have shown that hAECs inhibit cells of the innate and adaptive immune system, as shown by the inhibition of neutrophil and macrophage migration by secrete factors [8, 15] and reduction of both T and B cell proliferation [5, 16] in vitro.

The potential of hAECs for the treatment of MS has recently been highlighted by transplantation studies in experimental autoimmune encephalomyelitis (EAE) by us and others [17, 18], which links the amelioration of EAE with the capacity of hAECs to suppress inflammation. However, the mechanisms behind the suppression of disease are not well understood. It has also been claimed that hAECs are capable of homing to sites of inflammation [19], including the brain [20]. It is noteworthy that amnion has been used clinically for over 30 years for the treatment of dural defects [21], burns [22, 23], ocular surface disease [24], and other numerous eye diseases [25–27], thus establishing their strong safety profile. The current study extends on previous findings by showing that hAECs can significantly suppress in vitro T cell activation, proliferation, and cytokine production. When administered at the time of disease onset in a RR-EAE model, hAEC transplantation significantly attenuated disease through increasing the number of T regulatory cells, increasing the pool of peripheral naïve CD4+ T cells, and promoting a shift towards a Th2 dominant environment. This study demonstrates that hAECs are highly immunomodulatory and may have potential as a therapy for MS.

## Materials and methods

### Isolation of hAECs

All experiments using hAECs were performed with approval from the Monash Medical Centre Human Ethics Committee. Human AECs were isolated as previously described [13]. Briefly, placentae were obtained from women with uncomplicated pregnancies undergoing elective cesarean section at term. Women gave written, informed consent for the collection of their placenta. The amnion was manually stripped from the chorion, and the hAECs were enzymatically removed by two 1-h digestions using TrypZean (Sigma-Aldrich, St Lois, MO, USA). Following digestion, TrypZean was inactivated with Soybean trypsin inhibitor (Sigma-Aldrich) and the hAECs were collected by centrifugation (1800 g, 10 mins, RT). Live cell counts and viability were determined microscopically using trypan blue dye exclusion. Cells were cryopreserved using standard methods at 5 × 10^6^ cells/ml. To thaw, hAEC sample tubes were quickly removed from liquid nitrogen and placed directly into a 37 °C water bath until thawed. Samples were washed with ice cold media to remove DMSO, and cell counts and viability were determined. Cells were then transplanted in experimental mice or analyzed in in vitro assays.

### Flow cytometry

Phenotypic analysis by flow cytometry was performed by staining 0.5–3 × 10^6^ cells with primary antibodies for 20 min at 4 °C. Where appropriate cells were fixed and permeabilized for intracellular antibody staining according to the manufacturer’s instructions. All primary antibodies were purchased from BD Bioscience (San Jose, CA, USA). The relevant isotype control antibodies were used as negative controls. Cells were then washed with fluorescence-activated cell sorting (FACS) buffer [D-PBS containing 1 % FBS, 5 mM EDTA, 0.02 % (w/v) sodium azide (Sigma Aldrich)] by centrifugation at 300 g for 5 min at 4 °C. Data acquisition was performed using a FACSCalibur or FACSCanto II flow cytometer, and data were analyzed using Flowlogic Software (Inivai Technologies, Mentone, VIC, Australia).

### EAE induction and cell transplantation

All animal experiments were performed with approval from the Monash University School of Biomedical Sciences Animal Ethics Committee. EAE was induced in 10–12-week-old female NOD/Lt mice by subcutaneous injection of 75 μg extracellular domain of mouse recombinant myelin oligodendrocyte glycoprotein (rMOG; amino acid residues 1–117 of the mature protein) as previously described [28]. The mice also received 350 ng pertussis toxin (List Biological Laboratories Inc, CA, USA) intraperitoneally at the time of immunisation and 48 h later. For hAEC transplantation, cells were injected into the peritoneal cavity in a volume of 200 μl on the days indicated. Control mice received injections of equal volumes of phosphate buffered saline (PBS). The mice were monitored daily, and clinical scores were assigned according to an arbitrary scale as follows: 0 normal, 1 loss of tail tone only, 2 weakness in one or two hind limbs and abnormal gait, 3 hind limb paralysis, 4 hind limb paralysis and fore limb weakness, and 5 dead. The mice were humanely killed by carbon dioxide asphyxiation upon reaching a score of 4 or at the completion of the experiment.

### T cell proliferation assay and cytokine analysis

Proliferation assays using spleens from MOG TCR transgenic (2D2) mice and EAE mice were performed as previously described [29]. For experiments assessing the inhibition of T cell proliferation by hAECs, 50 μl of complete Roswell Park Memorial Institute (RPMI) medium or 50 μl of hAECs at hAEC to splenocyte ratios ranging from 1:5 to 1:625 were added to each well prior to the addition of splenocytes. Cells were incubated at 37 °C for 48 h and then 1 μCi/well [3H]-thymidine (Perkin Elmer, Waltham, MA, USA) was added for an additional 18 h of culture. Cells were harvested onto filter mats (Perkin Elmer) and incorporated radioactive nucleic acids counted using a Top Count Harvester (Packard Biosciences, Meriden, CT, USA).

Cytokine analysis was performed as previously described [29]. For experiments involving inhibition of cytokine production by hAECs, 500 μl of complete RPMI medium or 500 μl of hAECs at a hAEC to splenocyte ratio of 1:5 were added to each well prior to the addition of splenocytes. Culture supernatant was collected after 72 h, and quantitative analysis of cytokines was performed using a mouse Th1/Th2/Th17 cytometric bead array (CBA) kit (BD Bioscience) according to the manufacturer’s instructions. Data was acquired using a FACSCanto II flow cytometer (BD Bioscience) and analyzed using FCAP array software (Soft Flow Inc, Burnsville, MN, USA). The following cytokines were measured: IL-2, IL-4, IL-10, IL-17A, interferon gamma (IFN-γ), and tumor necrosis factor-alpha (TNF-α).

### Mixed lymphocyte reactions

Mononuclear cells from lymphoid tissues were prepared as above, and mixed lymphocyte reactions were performed. In brief, cells were seeded in 96-well, flat-bottom microtiter plates in triplicate. Splenocytes (2 × 10^5^) from C57BL/6 mice (effectors) were incubated with equal numbers of irradiated (20 Gy) Balb/c stimulators or hAECs. In experiments involving inhibition of allogeneic proliferation, hAECs were added as third-party cells at a hAEC to splenocyte ratio of 1:5 prior to the addition of splenocytes. Cells were incubated at 37 °C for 4 days and then 1 μCi/well [3H]-thymidine (Perkin Elmer) was added for an additional 18 h of culture. Cells were harvested onto filter mats (Perkin Elmer) and incorporated radioactive nucleic acids counted using a Top Count Harvester (Packard Biosciences).

### T cell phenotyping

Mononuclear cells from lymphoid tissue and the CNS were prepared as described previously [30], and all cell counts were performed using a Z2 Coulter cell and particle counter (Beckman Coulter, Miami, FL, USA). For naïve T cell staining, cells were stained with appropriately diluted cell surface antibodies purchased from BD Bioscience. Analysis of the regulatory T cells (Treg) was performed with 3 × 10^6^ cells using a FoxP3 staining set (eBioscience) according to the manufacturer’s instructions. For T helper cell phenotyping, cells were resuspended at 5 × 10^6^ cells/ml in complete RPMI medium containing 50 ng/ml PMA and 1 μg/ml ionomycin. Four microliters Golgistop (BD Bioscience) was also added for every 6 ml cell culture medium. Cells were seeded in 24-well plates at 5 × 10^6^ cells per well and incubated for 5 h at 37 °C with 5 % CO_2_. Cells were then harvested and counted, and intracellular cytokine staining was performed with 3 × 10^6^ cells using a mouse Th1/Th2/Th17 phenotyping kit (BD Bioscience), as per the manufacturer’s instructions. Cells were then analyzed using a FACSCanto II flow cytometer.

### Histological assessment of CNS tissue

For histological analysis of CNS tissues, the brain and spinal cord were dissected from the mice and fixed in 10 % formalin. Paraffin-embedded sections (5 μm) were cut and stained with hematoxylin-eosin (H&E), luxol fast blue (LFB), and Bielschowsky silver impregnation to assess inflammation, demyelination, and axonal damage, respectively, as previously described [29]. Microglia were identified using rabbit anti-ionized calcium-binding adaptor molecule 1 (IBA-1) antibody (Wako Pure Chemical Industries Ltd., Osaka, Japan), raised against synthetic peptide corresponding to the C-terminal of IBA-1. The antibody was diluted 1:500 in a PBS solution (0.1 mol/l, pH 7.4). All sections were treated with a secondary antibody (1:200; biotinylated anti-rabbit IgG antibody; Vector Laboratories, Burlingame, CA, USA), and staining was revealed using 3,3′-diaminobenzidine (Pierce Biotechnology, Rockford, IL, USA). The sections were viewed at a magnification of either × 100 or × 400 using light microscopy (Olympus BX-41, Japan). Immunoreactive cells were counted in three fields of view within a given region on two slides per animal to give six fields of view per region per animal. Activated and resting macrophages were identified by morphology as previously described [20]. Positive and negative control sections were included in each run.

### Statistical analysis

Results are expressed as the mean ± standard error of the mean (SEM). Statistical analysis was performed using Prism 5.03 (GraphPad Software). Experimental and control groups were compared using one-way ANOVA, and, where appropriate, Bonferroni post hoc analysis was used. A value of *P* < 0.05 was considered statistically significant.

## Results

### Phenotypic characteristics of hAEC

hAECs utilized in this study were obtained from a primary tissue source, not a cell line, and consequently do not have a defined immunological profile. Due to the importance of major histocompatibility complex (MHC) classes I and II molecules in controlling immune responses, the expression of immunological markers on freshly isolated hAECs from three separate amnion donors was examined. The immunological markers analyzed included HLA-ABC (MHC class I), HLA-DR (MHC class II), co-stimulatory markers CD80 and CD86, and the immune regulatory antigen, HLA-G [12, 31]. All hAECs, regardless of donor, did not express HLA-DR and co-stimulatory markers CD80 and CD86 (Table 1). In contrast, all hAECs were positive for HLA-ABC expression and expressed intermediate to high levels of HLA-G, consistent with a previous report [32].

**Table 1:** Surface marker expression of hAECs, as determined by flow cytometry

Surface marker	Donor 1	Donor 2	Donor 3
Immunological markers			
HLA-ABC	++	++	++
HLA-DR	−	−	−
CD80	−	−	−
CD86	−	−	−
HLA-G	++	++	+
Phenotypic markers			
EpCAM	++	++	++
CD90	+	+	+
CD73	−	−	−
CD105	−	−	−
CD45	−	−	−
CD34	−	−	−
Integrins			
Integrin-α2/CD49b	++	+	++
Integrin-α4/CD49d	−	−	−
Integrin-α6/CD49f	++	+	+
Integrin-β1/CD29	++	++	++
Ig superfamily CAMs			
ICAM-1/ CD54	−	−	−
VCAM-1/ CD106	−	−	−
Other			
CXCR4/ CD184	−	−	−
CD44	−	−	−

−, Not detected; +, low-intermediate expression (5–50 %); ++, high expression (>50 %)

Unlike mesenchymal stromal cells (MSCs), which are assessed using specific selection criteria [33], there are no current criteria for hAEC selection. However, if they are epithelial cells, then they must express epithelial cell adhesion molecule (EpCAM) and, conversely, be negative for the MSC markers CD90 and CD105 [13]. Therefore, further phenotypic characterisation analyzing the epithelial, mesenchymal, and hematopoietic composition of isolated hAECs was performed on all three amnion donor cells (Table 1). All cells were found to be positive for EpCAM and negative for mesenchymal (CD105, CD73) and hematopoietic markers (CD45 and CD34), as expected. Surprisingly, we found all donors expressed CD90, a MSC marker. While some investigators have also reported this [34], others have only reported CD90 expression in passaged and not primary cells [35].

In order to determine any other donor-specific differences that may influence the therapeutic potential of hAECs, extensive phenotypic profiling of adhesion molecules and integrins was assessed by flow cytometry (Table 1). Cells from all three donors were negative for the hyaluronic acid receptor CD44 and the immunoglobulin superfamily cell adhesion molecules (CAMs), including the intercellular adhesion molecule-1 (ICAM-1, CD54) and vascular cell adhesion molecule-1 (VCAM-1, CD106). The expression of integrins was similar between all hAEC donors, and all were negative for integrin-α4.

### Immunosuppressive properties of hAECs in vitro

In view of the important role that T cells play in EAE pathogenesis [36], the ability of hAECs to suppress T cell proliferation and activation was examined.

#### Effects of hAECs on proliferation and cytokine production of activated T cells

**Fig. 1:**
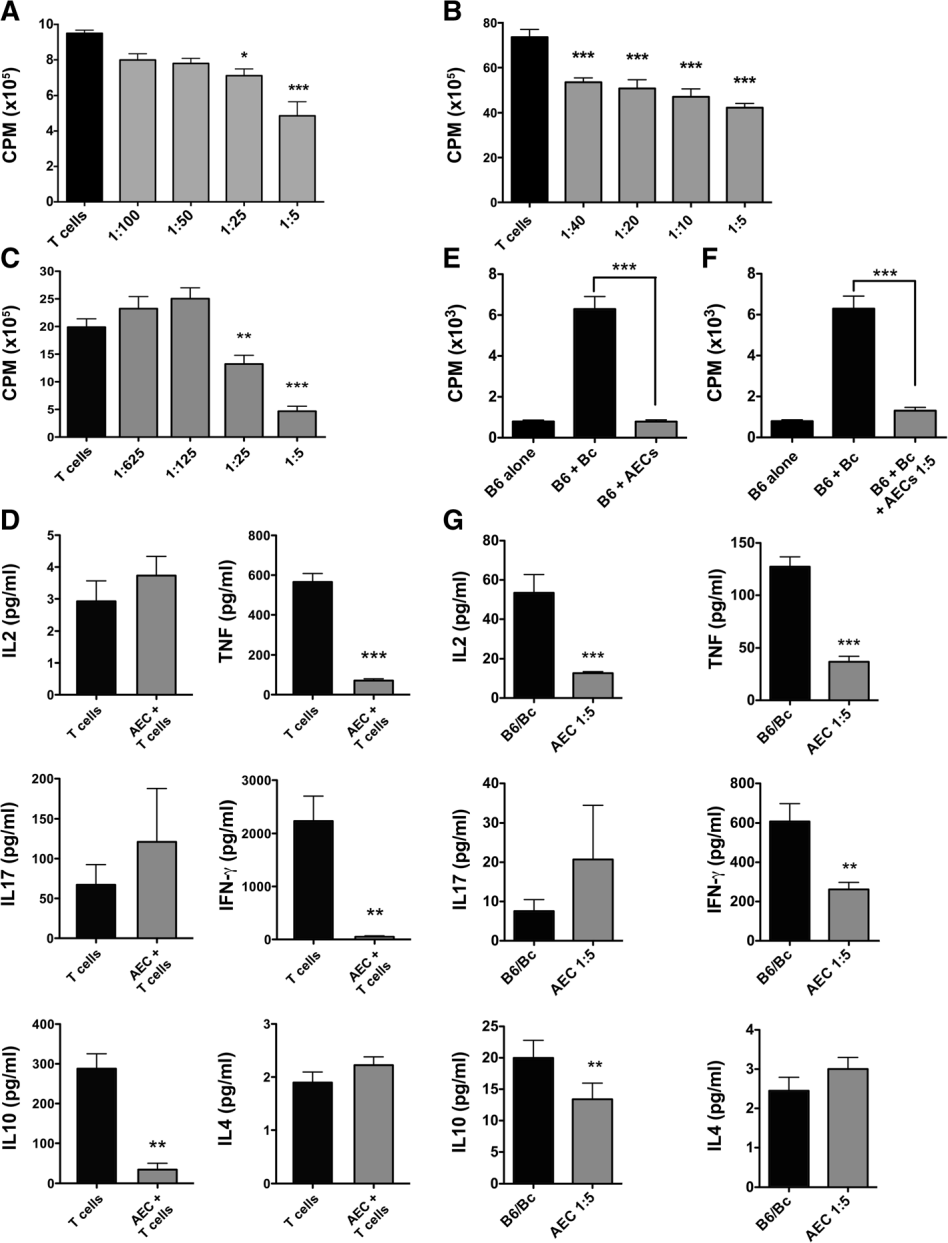
hAECs inhibit T cell responses and allogeneic and xenogeneic proliferation. Proliferative response of human PBMCs (**a**) or splenocytes from naïve C57BL/6 mice (**b**) stimulated with anti-CD3/CD28 antibodies in the presence or absence of hAECs at different hAECs to splenocyte ratios (*n* = 6 performed in triplicate, *P < 0.05, ***P < 0.001 compared to T cells). **c** Proliferative response of 2D2 splenocytes stimulated with 20 μg MOG35-55 in the presence or absence of hAECs at different ratios (*n* = 6 performed in triplicate, **P < 0.01, ***P < 0.001 compared to T cells). **d** Cytokine secretion profile in supernatant from co-cultures of hAECs and 2D2 splenocytes at 1:5 ratio (*n* = 5, **P < 0.01 ***P < 0.001 compared to T cells). **e** Proliferative response of C57BL/6 splenocytes co-cultured with hAECs (*n* = 4 performed in triplicate, ***P < 0.0001 compared to B6 + Bc). **f** Proliferative response of C57BL/6 splenocytes co-cultured with irradiated Balb/c splenocytes in the absence or presence of hAECs at hAEC to C57BL/6 T cell ratio of 1:5 (*n* = 4 preformed in triplicate, ***P < 0.0001 compared to B6 + Bc). **g** Cytokine secretion profile in supernatant from MLR cultures with hAECs. (*n* = 4, *P < 0.05,**P < 0.01, ***P < 0.001 compared to B6 + Bc)

For assessment of the suppressive ability of hAECs, we implemented a co-culture assay that incorporated hAECs together with human peripheral blood mononuclear cells (PBMCs) or mouse splenocytes stimulated with anti-CD3/CD28 (Fig. 1a, b, respectively). At high concentrations of hAECs, 1:5 (*P* < 0.001) and 1:25 (*P* < 0.05), human PBMC proliferation was significantly suppressed compared to PBMC stimulation alone. The proliferative response of splenocytes from naïve mice to anti-CD3/CD28 stimulation was significantly reduced at all hAEC concentrations tested (*P* < 0.001). Next, we assessed the ability of hAECs to suppress antigen-specific activated splenocytes from the 2D2 mice. These transgenic mice are genetically modified so that most of their CD4+ T cells recognise MOG35-55, resulting in a highly proliferative MOG-specific T cell response. hAECs were added at differing ratios to 2D2 splenocytes, and co-cultures were stimulated with 20 μg/ml MOG35-55. As shown in Fig. 1c, hAECs significantly suppressed the proliferation of MOG-specific T cells at hAEC to splenocyte ratios of 1:5 (*P* < 0.0001) and 1:25 (*P* < 0.01) by approximately 75 and 35 %, respectively, as compared to T cells alone. No significant differences were observed at 1:125 and 1:625. These data indicate that hAECs were able to suppress the proliferation of autoantigen-specific T cells in a dose-dependant manner. Supernatants were collected from MOG35-55 stimulated 2D2 splenocytes co-cultured with hAECs at a ratio of 1:5, and cytokines were quantified by CBA. A significant reduction in the pro-inflammatory cytokines IFN-γ (*P* < 0.01) and TNF-α (*P* < 0.001) as well as the anti-inflammatory cytokine IL-10 (*P* < 0.01) was observed in the presence of hAECs (Fig. 1d), while no change in IL-2, IL-17A, or IL-4 was detected (Fig. 1d).

#### Effect of hAECs on allogeneic T cell proliferation and cytokine production

We next analyzed the in vitro suppressive effects of hAECs in a one-way MLR. Co-cultures were established using C57BL/6 responder splenocytes and irradiated Balb/c stimulator splenocytes, with hAECs added as third-party cells at a ratio of 1:5. In contrast to the highly proliferative allogeneic reaction induced by Balb/c stimulators (*P* < 0.001), human-derived AECs did not stimulate a xenogeneic T cell response when cultured with C57BL/6 responders (Fig. 1e) and no difference in T cell proliferation was observed in the presence of hAECs when compared to C57BL/6 cells alone. Notably, hAECs were able to significantly suppress allogeneic immune responses between splenocytes from C57BL/6 and Balb/c mice (*P* < 0.001; Fig. 1f), the proliferation being reduced by 80 %. We next examined how hAECs exerted their suppressive effect on T cells by analyzing cytokine production in culture supernatants. As shown in Fig. 1g, the addition of hAECs at 1:5 significantly reduced the production of IL-2 (*P* < 0.001), IFN-γ (*P* < 0.01 and *P* < 0.05, respectively), TNF-α (*P* < 0.001), and IL-10 (*P* < 0.01). No changes were observed for IL-17A or IL-4.

### Inhibition of activation and maintenance of a naïve phenotype of T cells co-cultured with hAECs

**Fig. 2:**
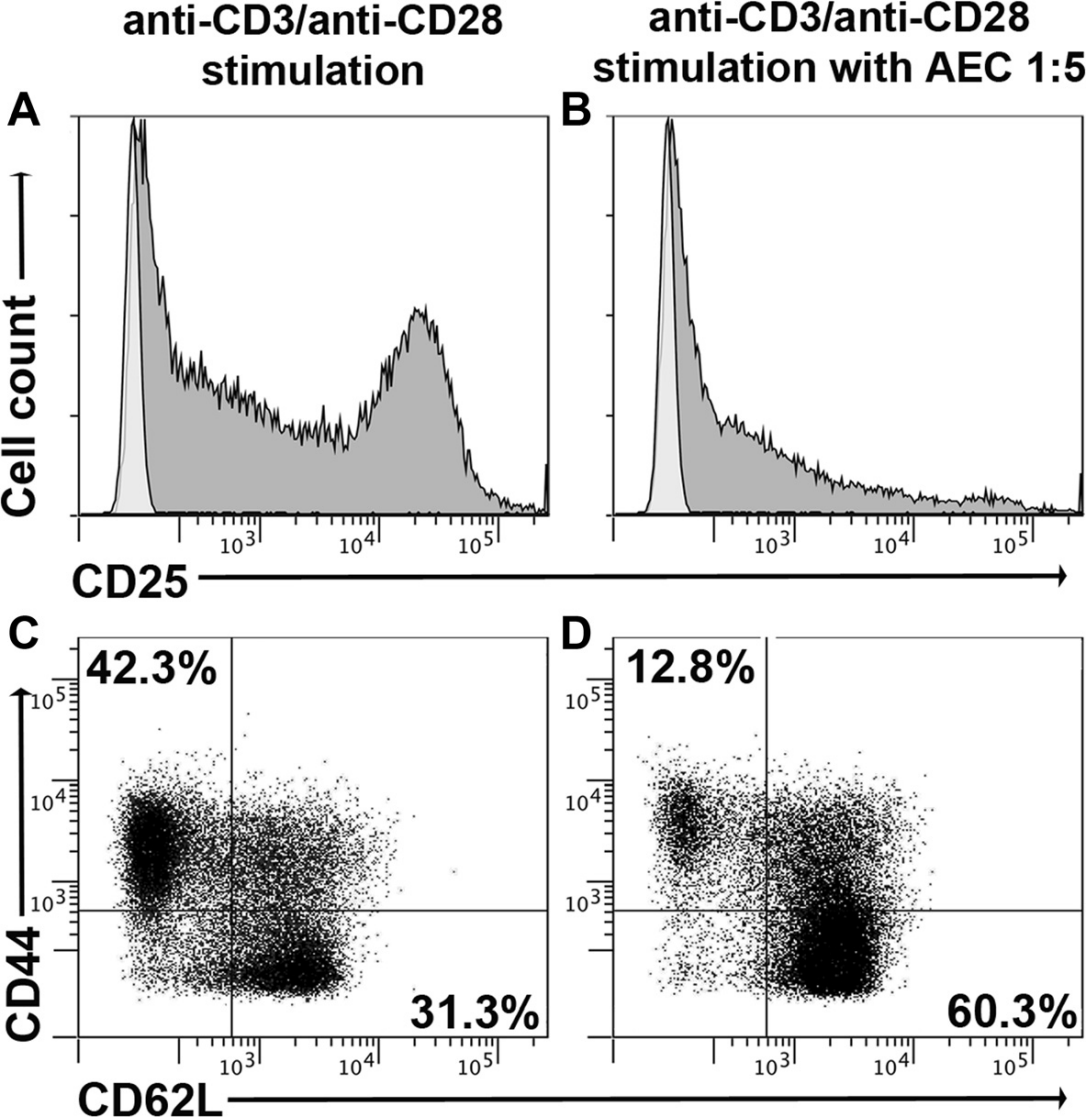
hAECs inhibit upregulation of T cell activation markers and retain T cells in a naïve phenotype. Naïve CD4+ T cells were stimulated with anti-CD3/CD28 in the presence or absence of hAECs for 5 days and then analyzed by flow cytometry. **a**, **b** Representative profiles of CD25 expression on stimulated CD4+ T cells. **c**, **d** Representative profiles of CD44 and CD62L expression on stimulated CD4 + CD25+ T cells. CD62LhiCD44lo denotes naïve T cells, CD62LloCD44hi denotes memory cells. Data are representative of two independent experiments with *n* = 3 mice

We next assessed the direct effect of hAEC co-culture on the expression of the activation marker CD25 on T cells. For this, naïve CD4+ T cells (CD62LhiCD44lo) were isolated from the spleens of the C57BL/6 mice and stimulated with anti-CD3/anti-CD28 in the presence or absence of hAECs (Fig. 2). After 5 days of culture, T cells were collected and examined for the expression of CD25, CD62L, and CD44 using flow cytometry. Stimulation of naïve T cells alone resulted in the upregulation of CD25 (Fig. 2a), with 42.3 % of CD4+ T cells exhibiting a memory phenotype (CD62LloCD44hi) and 31.3 % remaining in a naïve state (Fig. 2c). In contrast, in the presence of hAECs, there was a large reduction in the upregulation of CD25 (Fig. 2b) and significantly less CD4+ T cells acquired a memory phenotype. Indeed, only 12.8 % of T cells expressed a memory phenotype and 60.3 % retained their naïve phenotype (Fig. 2d).

### Administration of hAECs ameliorates relapsing-remitting EAE in a dose-dependant manner

**Fig. 3:**
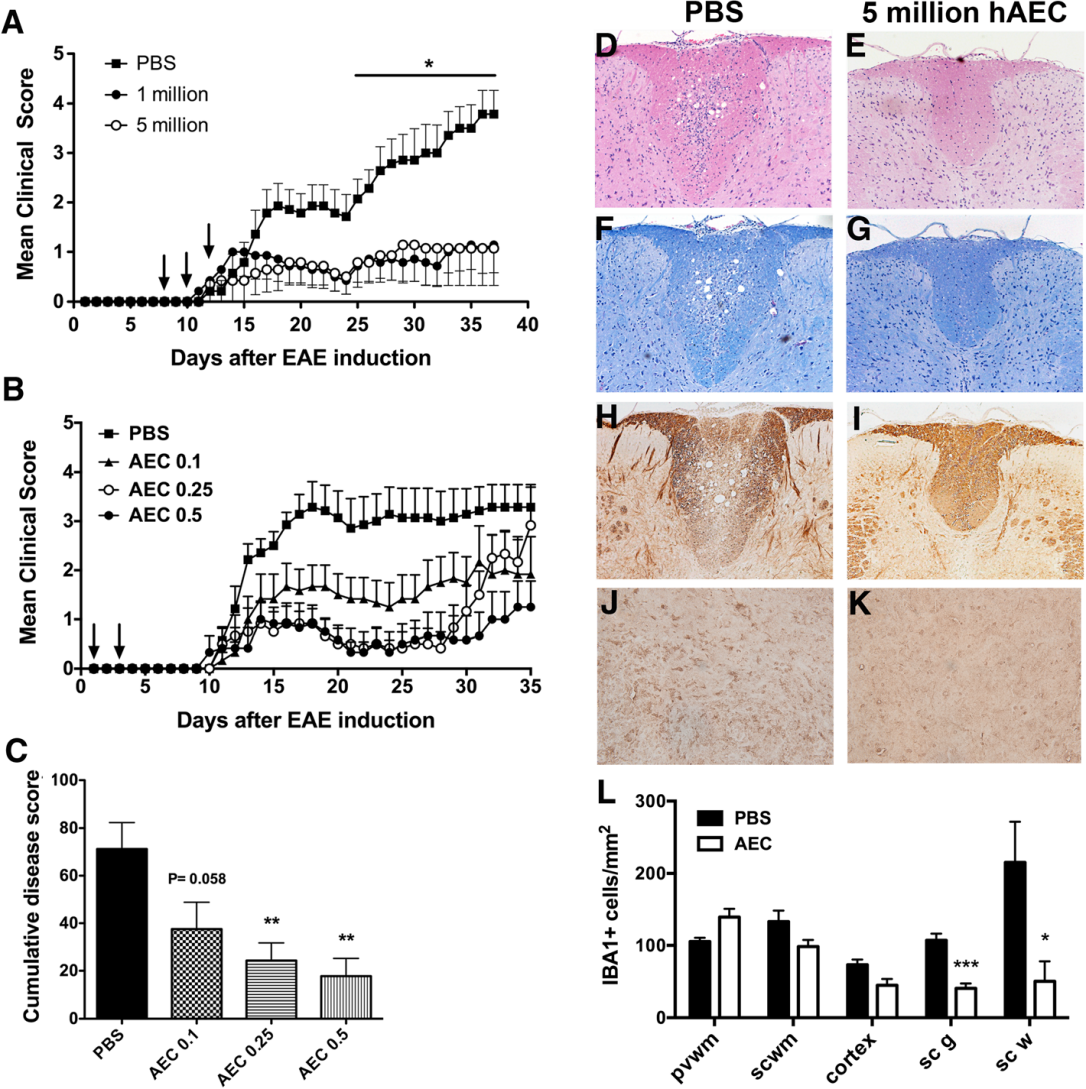
Administration of hAECs on day 8, 10, and 12 suppresses the development of RR-EAE. **a** Clinical scores of NOD/Lt mice injected i.p. with one or five million hAECs or PBS on day 8, 10, and 12 (indicated by arrows) (*n* = 7, *P < 0.05 compared to PBS). Clinical scores (**b**) and cumulative disease score (**c**) of NOD/Lt mice injected i.p. with 0.1, 0.25, or 0.5 million hAECs or PBS on day 1 and 3 (indicated by arrows) (*n* = 6–7, **P < 0.01 compared to PBS). Representative spinal cord sections from PBS control mice stained with H&E (**d**), LFB (**f**), or Bielschowsky silver stain (**h**) or mice that received five million hAECs stained with H&E (**e**), LFB (**g**), or Bielschowsky silver stain (**i**) to assess inflammation, demyelination, and axonal damage, respectively. Magnification × 100. IBA-1+ immunoreactivity in the grey matter of spinal cord sections from PBS controls (**j**) or mice that received five million hAECs (**k**). Magnification × 400. (**l**) Total number of IBA-1+ cells/mm^2^ in different regions of the CNS in PBS controls and hAEC-treated mice (*P < 0.05 compared to PBS)

In vitro data indicated that hAECs are potent suppressors of immune cell activation and proliferation. To test whether hAECs could provide a therapeutic benefit in an inflammatory setting, we used a relapsing-remitting EAE mouse model. Following EAE induction, we injected either one or five million hAECs at the onset of disease on days 8, 10, and 12. A significant amelioration of EAE severity was seen in the daily clinical scores following the administration of both doses of hAECs (*P* < 0.05; Fig. 3a), as compared to the PBS group. Importantly, the PBS control mice relapsed at day 24, while hAEC-treated mice did not relapse for the duration of the study, suggesting that cell administration also prevented relapse in these mice. The incidence of disease in the PBS control group was 100 %, while hAEC administration decreased the incidence to 57 and 42 % for the groups receiving one or five million cells, respectively (Table 2). There was no significant difference in the day of onset of clinical signs between the groups. However, the maximum disease scores and cumulative disease scores were significantly (*P* < 0.05) reduced in both the hAEC cell treatment groups compared to the PBS group.

**Table 2:** Clinical outcome of relapsing-remitting-EAE mice treated with hAECs

	1 × 10^6^ hAECs	5 × 10^6^ hAECs	PBS
Disease incidence	4/7	3/7	7/7
Day disease onset	17.25 ± 5.3	15.7 ± 2.0	16.7 ± 1.6
Death severe disease	1/7	1/7	3/7
Maximum score	1.9 ± 0.6*	1.3 ± 0.7*	3.8 ± 0.5
Cumulative score	24.9 ± 10.2*	20.8 ± 12.0*	57.3 ± 9.79

*P < 0.05 compared to PBS

**Fig. 4:**
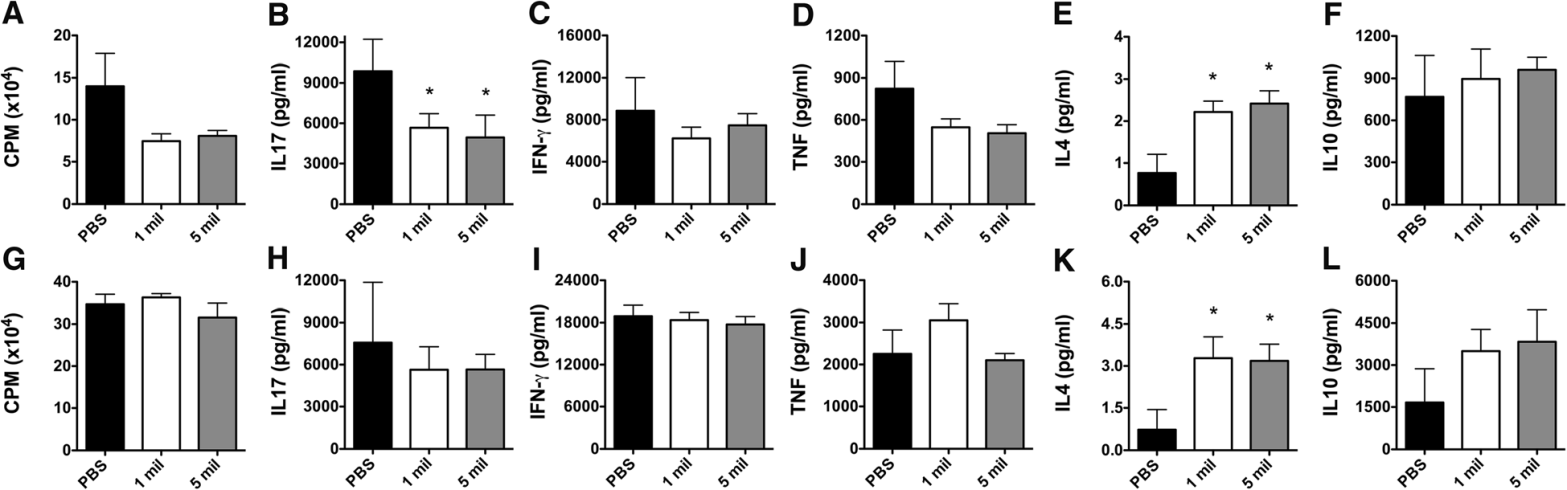
T cell responses in RR-EAE mice following hAEC administration. Mononuclear cells were isolated from the spleen of NOD/Lt mice at day 37. Proliferative response of splenocytes stimulated with rMOG (**a**) or anti-CD3/CD28 (**g**). **b**–**f** Cytokine secretion profile in supernatant from rMOG stimulated splenocyte cultures. **h**–**l** Cytokine secretion profile in supernatant from anti-CD3/CD28 stimulated splenocyte cultures (*n* = 6 performed in triplicate, *P < 0.05 compared to PBS)

Neuropathological evaluation of CNS tissues from the PBS group showed extensive inflammatory lesions, which were characterised by infiltration of mononuclear inflammatory cells in H&E stained sections (Fig. 3d). Luxal fast blue (Fig. 3f) and Bielschowsky silver staining (Fig. 3h) also showed marked myelin loss and severe axonal injury, respectively, particularly around lesioned tissue within the dorsal column of the spinal cord. Analysis of histological sections from the mice transplanted with five million cells revealed little cellular infiltration with minimal demyelination and axonal damage (Fig. 4e, g, i). Furthermore, we assessed IBA-1+ immunoreactivity, which stains microglia and macrophages, within different regions of the CNS. A significant decrease in IBA1+ cells was found in both the white and gray matters of the spinal cord, following hAEC administration compared to the PBS controls (Fig. 3j, k, respectively). No difference was observed in the cortex, periventricular, or subcortical white matter (Fig. 3l). Collectively, administration of hAECs into RR-EAE results in amelioration of both clinical and neuropathological signs of disease.

As hAEC administration was effective in ameliorating RR-EAE, we titrated the number of cells administered to determine whether suppression of EAE by hAECs was dose-dependant. To that effect, hAECs were administered i.p. at three different concentrations, 0.5, 0.25, and 0.1 million. A dose-dependant attenuation of EAE severity was observed with an increasing cell dose (Fig. 3b). The two highest doses of hAECs significantly reduced the cumulative disease score (*P* < 0.01), a trend that was almost significant following administration of only 0.1 million hAECs (*P* = 0.058; Fig. 3c). Administration of 0.5 million hAECs significantly reduced the maximum disease score compared to the PBS control group (*P* < 0.05; Table 3). No significant differences were observed between the groups in terms of the disease incidence, day of disease onset, or death due to severe disease (Table 3).

**Table 3:** Clinical results from hAEC dose titration

	hAEC 0.1	hAEC 0.25	hAEC 0.5	PBS
Disease incidence	5/6	5/6	5/6	7/7
Day disease onset	12.8 ± 0.6	17.0 ± 3.7	14.0 ± 1.3	12.0 ± 0.4
Death severe disease	1/6	2/6	0/6	2/7
Maximum score	2.3 ± 0.7	3.1 ± 0.8	1.8 ± 0.5*	3.6 ± 0.4
Cumulative score	37.6 ± 11.3	24.3 ± 7.8**	17.8 ± 7.5**	71.2 ± 11.1

*P < 0.05, **P < 0.01, compared to PBS

### Effect of hAEC administration on peripheral immune responses in RR-EAE

Having shown that hAEC administration ameliorates RR-EAE disease severity in a dose-dependant manner, the effect on peripheral immune responses was then examined. Splenocytes from the treated mice were isolated and stimulated in vitro with either rMOG or anti-CD3/CD28 for 72 h. In comparison to the PBS control group, a trend towards decreased MOG-specific proliferation was seen in the hAEC-treated groups receiving either one or five million hAECs (Fig. 4a). This was accompanied by a significant decrease in IL-17A (*P* < 0.05; Fig. 4b) and TNF-α secreted in splenocyte cultures from the mice that received hAECs (Fig. 4d). In contrast, a significant increase in the secretion of the anti-inflammatory cytokine IL-4 was observed in the mice that received hAECs (*P* < 0.05; Fig. 4e). No difference in MOG-specific secretion of IFN-γ (Fig. 4c) or IL-10 (Fig. 4f) was observed between the groups. It is noteworthy that this cytokine profile of decreased IL-17A and increased IL-4 observed following hAEC administration was also found to be dose-dependant (data not shown).

The effect of hAEC administration on non-specific peripheral immune responses was also determined by stimulating splenocytes with anti-CD3/CD28. No reduction in non-specific T cell responsiveness (Fig. 4g) or production of cytokines such as IL-17A (Fig. 4h), IFN-γ (Fig. 4i), and TNF-α (Fig. 4j) was observed following the transplantation of hAECs. Importantly, a significant increase in IL-4 secretion was observed in both the hAEC-treated groups compared to PBS (*P* < 0.05; Fig. 4k). Collectively, these data suggest that T cells had acquired a Th2-biased phenotype in the mice that received hAECs.

### hAEC administration increases the number of T regulatory cells and naïve CD4+ T cells in the periphery

**Fig. 5:**
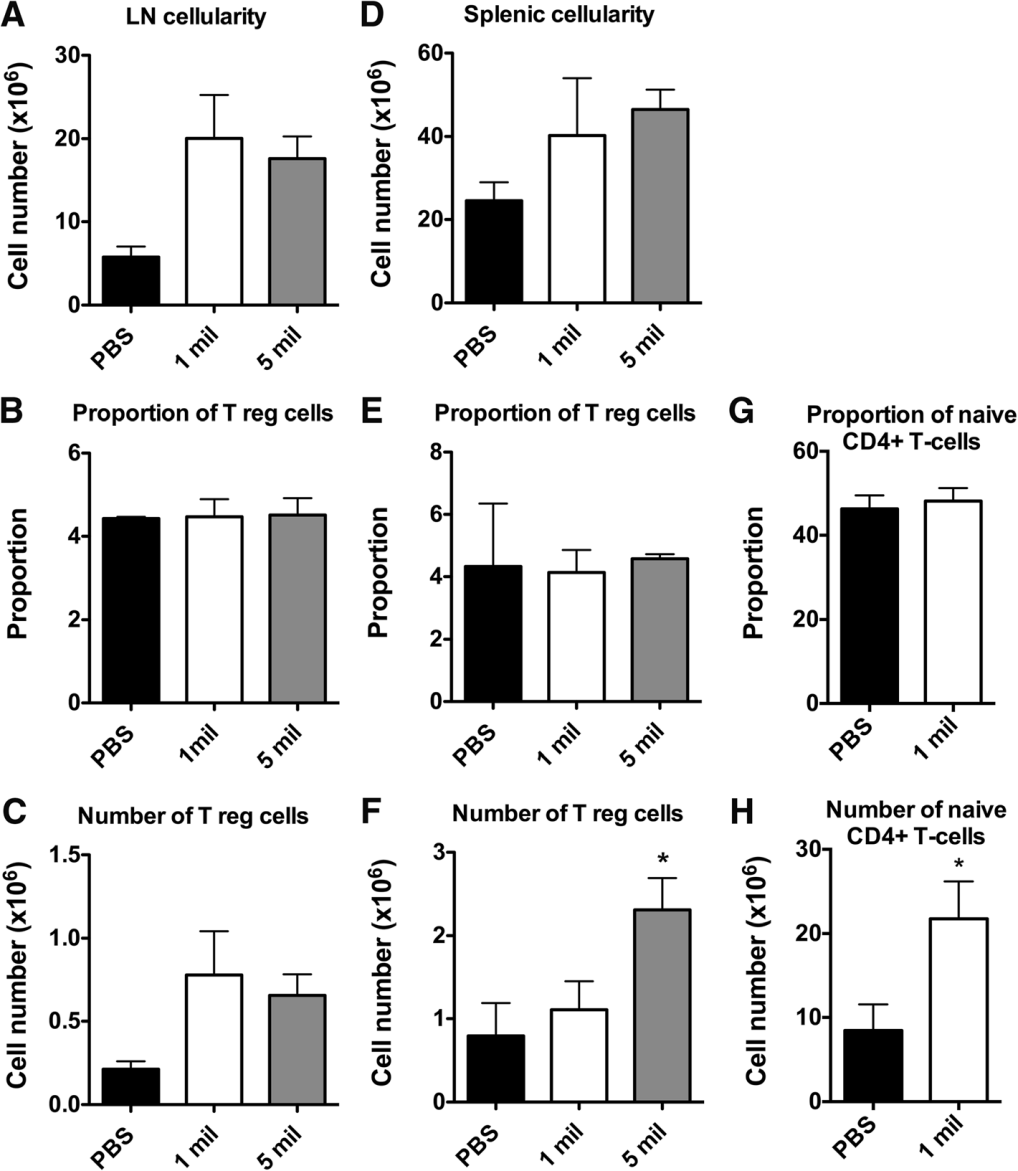
Administration of hAECs increases the total number of Tregs and naïve CD4+ T cells in the periphery. Mononuclear cells were isolated from the lymph nodes and spleen at day 37, and the proportion and number of Tregs or naïve CD4+ T cells was analyzed by flow cytometry. **a** Total cell number in the lymph nodes. **b** Proportion of gated lymphocytes that are CD4 + CD25 + Foxp3+ Tregs in the lymph nodes. **c** Total Treg numbers in the lymph nodes. **d** Total cell number in the spleen. **e** Proportion of gated lymphocytes that are CD4 + CD25 + Foxp3+ Tregs in the spleen. **f** Total Treg numbers in the spleen. **g** Proportion of CD4+ that are naïve (CD62LhiCD44lo) T cells in the spleen. **h** Total number of CD4+ naïve (CD62LhiCD44lo) T cells in the spleen. (*n* = 6, *P < 0.05 compared to PBS)

To gain a better understanding of the mechanism by which protection against EAE was afforded by hAECs, the proportions and total numbers of CD4 + CD25 + FoxP3+ Tregs were examined in the lymph nodes and spleen. A trend towards decreased total cell numbers was observed in the lymph nodes (Fig. 5a) and spleen (Fig. 5d) of the PBS control mice compared to both the hAEC-treated groups. No change in the proportion of Tregs was detected between the groups in the lymph nodes (Fig. 5b); however, a trend towards increased total Treg numbers was observed in the lymph nodes of both the hAEC-treated groups (Fig. 5c). Similarly, no change in the proportion of Tregs was observed in the spleen (Fig. 5e); however, a significant increase in total Treg numbers was observed in the spleen of the mice that received five million hAECs compared to the PBS controls (*P* < 0.05; Fig. 5f). Moreover, when the presence of naïve CD4+ T cells in the spleen was investigated, no change in proportion could be found between the groups (Fig. 5g). However, a significant increase in the total number of naïve CD4+ T cells was observed in the mice that received one million hAECs compared to the PBS controls (*P* < 0.05; Fig. 5h). Based on these results, the protection afforded by hAECs in the EAE mice appeared to be mediated, in part, by an increase in the number of Tregs and naïve CD4+ T cells in peripheral lymphoid organs.

### Effect of hAEC administration on CD4+ T helper cell subsets

**Fig. 6:**
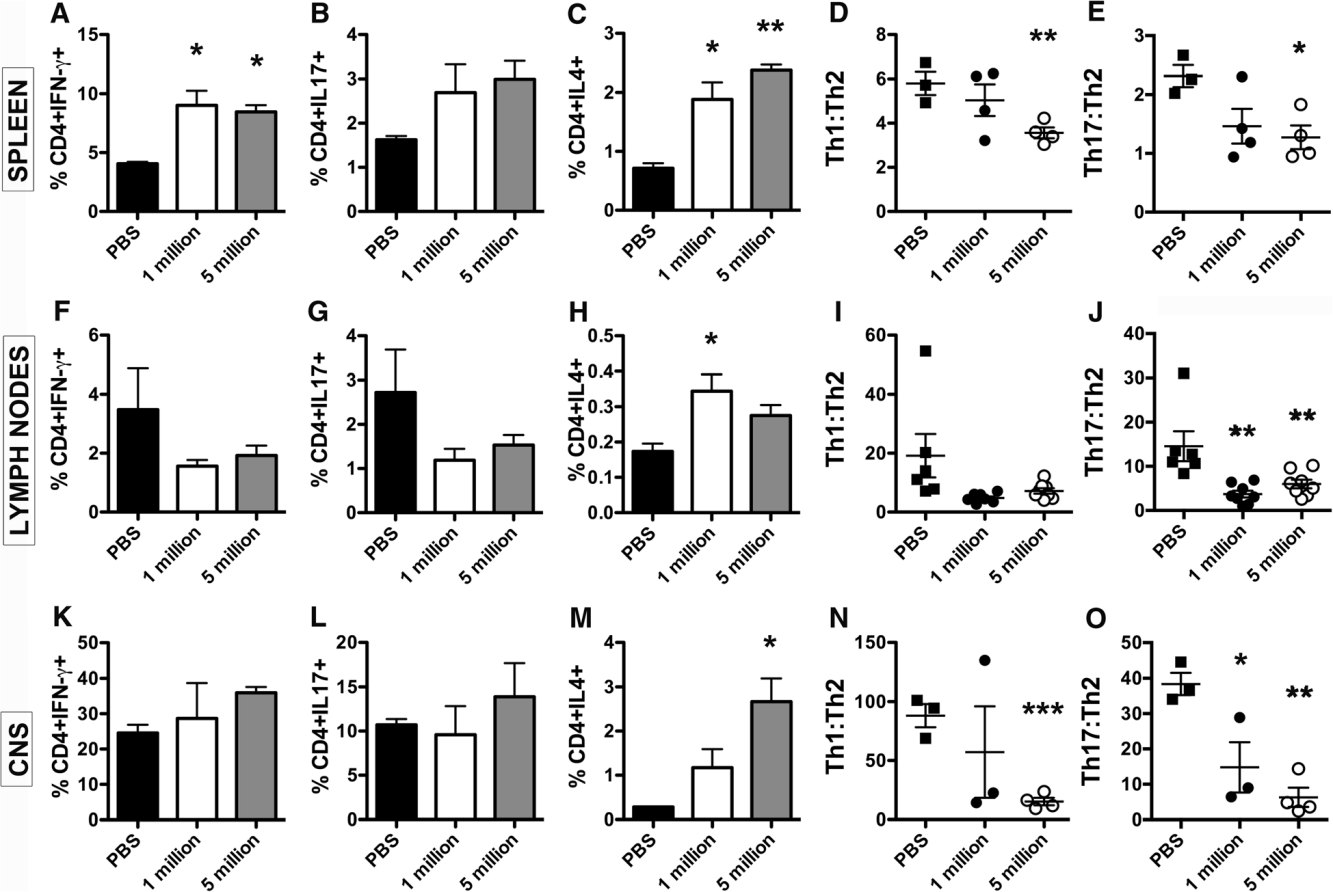
Administration of hAECs increases Th2 cells in the CNS and shifts the inflammatory profile towards an anti-inflammatory environment. Mononuclear cells were isolated from NOD/Lt mice at day 37 and the phenotype of T helper cells was assessed by flow cytometry. Th1 (CD4 + IFN-γ+), Th17 (CD4 + IL-17+), and Th2 (CD4 + IL-4+) cell proportions and the Th1 to Th2 and Th17 to Th2 ratios were determined in the spleen (**a**–**e**), lymph nodes (**f**–**j**), and CNS (**k**–**o**) (*n* = 6, *P < 0.05, **P < 0.01, ***P < 0.001)

Using IFN-γ, IL-4, and IL-17A as biomarkers of Th1, Th2, and Th17 cells, respectively, we examined the percentage of these CD4+ T helper cells in the periphery and CNS of the hAEC-treated mice. Within the spleen, there was a significant increase in the proportion of CD4 + IFN-γ + T cells (*P* < 0.05; Fig. 6a) compared to the PBS controls and a trend towards increased proportions of CD4 + IL17-A+ T cells (Fig. 6b). The proportion of CD4 + IL-4+ Th2 cells was found to be significantly increased in the spleens of the mice receiving hAECs at doses of one million (*P* < 0.05) and 5 million (*P* < 0.01) cells, compared to the PBS control group (Fig. 6c). The increase in Th1 and Th17 cells was not observed in the lymph nodes (Fig. 6f, g, respectively), although a Th2 shift, indicated by a significant increase in the proportion of CD4 + IL-4+ cells, was observed in the group of mice receiving one million AECs (*P* < 0.05; Fig. 6h). Finally, in the CNS, no differences were observed in the proportions of CD4 + IFN-γ + and CD4 + IL-17+ T cells between the groups (Fig. 6k, l, respectively). However, there were increases in the proportion of CD4 + IL-4+ T cells in the mice that received hAECs, with figures reaching significance in the mice transplanted with five million hAECs (*P* < 0.05; Fig. 6m), compared to the PBS control group.

Recent evidence has suggested that both Th1 and Th17 cells play a role in EAE pathogenesis. We therefore determined the ratio of Th1 to Th2 and Th17 to Th2 cells within the spleen, lymph nodes, and CNS. Within the spleen, the Th1 to Th2 (*P* < 0.01; Fig. 6d) and Th17 to Th2 (*P* < 0.05; Fig. 6e) ratios were significantly decreased in the mice that received five million hAECs compared to PBS, suggesting a shift towards an anti-inflammatory Th2 phenotype. This finding is in line with our previous results that found increased secretion of IL-4 in spleen cultures (Fig. 4e, k). Analysis of the Th1 to Th2 ratio in the lymph nodes trended towards a reduction in the Th1 to Th2 ratio in both the hAEC-treated groups compared to the PBS control (Fig. 6i), and the Th17 to Th2 ratio in the lymph nodes was significantly reduced following hAEC administration (*P* < 0.01; Fig. 6j). Upon examination of the Th1 to Th2 (Fig. 6n) and Th17 to Th2 (Fig. 6o) ratios in the CNS, a highly significant decrease in both ratios was observed between the group receiving five million hAECs and the PBS control group (*P* < 0.001 and *P* < 0.01, respectively). Collectively, these data indicate an equilibration of the Th1 to Th2 and Th17 to Th2 balance and a bias towards a Th2 phenotype within the peripheral immune organs and the CNS following hAEC transplantation.

## Discussion

hAECs are a novel source of stem cells that can be obtained in large quantities [13] and possess potent immunosuppressive properties [5, 15]. In the current study, we examined whether the administration of hAECs in a RR-EAE model could modulate the immune response and suppress MOG-induced EAE development. Our results show that transplantation of hAECs at the time of disease onset significantly ameliorates RR-EAE by inhibiting pathogenic T cell responses, namely Th17 and Th1 cells within the periphery and CNS. We propose that this effect is mediated by an increase in the number of peripheral T regulatory cells, an increase in the pool of naïve CD4+ T cells, and a shift towards a Th2 dominant environment.

Given that hAECs are routinely isolated from different donors, it is important for their future clinical application that donor-specific variations are assessed. We confirmed that hAECs do not express HLA class II antigen (HLA-DR) or co-stimulatory factors CD80 and CD86 [12], and no variation between donors was noted. However, we did observe expression of HLA class I antigens in cells from all of our donors, which is in contrast to previous reports [9, 37] and may lead to an increased risk of transplant rejection. The expression of HLA-G, a non-classical class I antigen, is most commonly expressed on placental tissues [38, 39], including amnion [32]. We found that there was variation in HLA-G expression between donors. While all were positive, some had threefold higher expression than others. It has previously been reported that preterm amnion expresses less HLA-G than term amnion [32]. It is possible that HLA-G expression is related to proximity to the timing of birth. Furthermore, upon analysis of CD90, we found discrepancies to previous literature that have stated that hAECs are negative for this marker [13]. In this current study, we did not directly compare the in vivo efficacy between the different donors, but given the differences in HLA-G and CD90 expression, these future studies are warranted. The phenotypic differences we observed between the donors in this study suggest that the pre-screening of amnion donors may be required in order to identify those most suitable for use in specific clinical applications, such as the treatment of MS patients.

As shown here, hAECs posses potent immunomodulatory properties capable of suppressing antigen-specific, non-specific, and allogeneic T cell responses in vitro, including human PBMC proliferation. While it has previously been shown that hAECs do not stimulate allogeneic proliferation when cultured with PBMCs [12], our observations further demonstrate that these cells do not elicit a xenogeneic response from mouse splenocytes. The immune suppressive activity of hAECs in vitro was associated with a significant decrease in Th1 cytokines, including IFN-γ and TNF-α. It is interesting to note that hAECs did not influence secretion of IL-17A and IL-4 in in vitro assays, while in vivo, hAEC treatment led to decreased IL-17A and increased IL-4 secretion. This may be explained by differences in mouse strain (C57BL/6 versus NOD/Lt) and stimulating antigen (MOG35-55 versus recombinant MOG protein) used in the in vitro and in vivo experiments. While these results are not directly comparable, it is noteworthy that hAECs suppress proliferation and pro-inflammatory cytokines in both settings, thus demonstrating their broad immunosuppressive properties. In examining the in vitro suppressive effect of hAECs, we show here for the first time that hAECs restrain naïve CD4+ T cells from developing into an activated phenotype. This was demonstrated by inhibition of the upregulation of two markers, the T cell activation marker CD25, and the adhesion receptor CD44. This is important in the context of MS pathogenesis, since MS patients have more myelin-reactive effector/memory T cells compared to healthy controls [40]. In the EAE mice, CD44 has been shown to be involved in both the differentiation of Th1/Th17 cells [41] and the extravasation of effector/memory CD62LloCD44hi T cells into the CNS [42, 43].

The ability of a single dose of two million hAECs to ameliorate EAE has previously been shown in the C57BL/6 mice [18], and our earlier work has also demonstrated that a single dose of one million hAECs can suppress EAE in the NOD/Lt mice [17]. Results presented here extend these findings by demonstrating that low-dose (1 × 10^6^) and high-dose (5 × 10^6^) hAECs injected intraperitoneally were effective in significantly ameliorating clinical and pathological signs of RR-EAE in the NOD/Lt mice. We also demonstrate for the first time that hAECs ameliorate EAE in a dose-dependant manner and importantly, as little as 100,000 hAECs suppressed clinical signs of the disease. We have previously investigated the efficacy of different sources of MSCs [29] and neural stem cells (NSCs) [28] in EAE and have never observed significant amelioration of disease when less than one million cells were transplanted. While not directly comparable, the current results suggest that hAECs are more powerful in suppressing EAE than MSCs and NSCs.

Liu et al. [18] have previously suggested that hAECs work via a Th2 shift based on the single observation that IL-5 was increased in the EAE mice treated with hAECs. However, no change in other cytokines, including IL-4, IL-17, IFN-γ, TNF-α, or IL-10, was reported. Moreover, no investigation was performed on the individual T helper cell phenotypes. In that study, EAE was induced in the C57BL/6 mice resulting in chronic progressive paralysis, a model that displays different immune responses compared to the relapsing-remitting EAE in the NOD/Lt mice [44]. Furthermore, Liu et al. [18] transplanted cells intravenously, whereas we delivered cells into the intraperitoneal cavity. While we cannot exclude the possibility that the variability seen in the cytokine secretion between these two studies is due to the strain of mice used for EAE, as well as the route of hAECs injection, it is noteworthy that we have previously shown that both administration routes are effective for reducing clinical signs [29]. In our study, we unequivocally show that, together with the decrease in clinical scores, we observed a significant decrease in MOG-specific secretion of IL-17A and a significant increase in IL-4, a Th2 cytokine that has been associated with spontaneous EAE recovery [45] and reduction in disease severity [46]. Importantly, the decreased secretion of IL-17A was observed in spite of the significant increase in the proportion of Th17 and Th1 cells within the spleen. Collectively, these data suggest that the increased proportion of Th1 and Th17 cells in the spleens of the hAEC-treated mice produce less pro-inflammatory mediators and are under the tight regulatory control of either Th2 or other regulatory cell types. This was verified by examining the Th1 to Th2 and Th17 to Th2 ratios, whereby the mice receiving five million hAECs displayed a significant Th2 shift.

Regulatory T cells play a key role in controlling immune responses and protect against the development of EAE [47]. In our study, increased numbers of Treg cells were observed in the peripheral lymphoid tissues of the mice that received hAECs. Using a model of fibrotic lung injury, we have recently shown that hAECs induce Treg polarisation and these Tregs were critical for macrophage polarisation and subsequent injury resolution [48]. Taken together, it is clear that hAECs have the ability to influence Tregs and this functional property is critical to their overall therapeutic efficacy. Studies using other placental-derived cells have found similar effects with increased Treg polarisation and downregulation of Th1 and Th17 responses in normal PBMCs and collagen reactive T cells from arthritis patients [49, 50]. Given the plasticity of CD4 cell subsets, further studies are required to define how hAECs regulate the differentiation of T helper cells and Tregs. We showed in vitro that hAECs had the ability to restrain naïve CD4+ T cells from acquiring a memory phenotype upon activation. Therefore, in our in vivo studies, we examined the naïve CD4+ T cell pool and similarly found a significant increase in the number of naïve CD4+ T cells in the mice treated with the low dose of one million hAECs compared to the PBS controls. This demonstrates that hAECs also have the ability to suppress the activation of T cells in vivo, which has not been shown previously.

While our findings show that hAECs act via modulation of peripheral immune responses, administration of hAECs also modulates the immune response directly within the CNS. This was demonstrated by a decrease in microglia and macrophages within both the gray and white matters of the CNS. Not only were there less overall numbers of macrophages and microglia, but the activation state of microglia was decreased, with more resting microglia present following hAEC administration (data not shown). Furthermore, an increase in the proportion of CD4 + IL-4+ T cells directly within the CNS was also observed in the mice that received hAECs. Increased IL-4 levels within the CNS of the EAE mice have been associated with decreased demyelination and axonal pathology [51] as well as promoting the recruitment of Tregs [52]. Th2 cytokines have also been found to have a positive effect on neuronal cultures by upregulating arginase, which can aid in neuroprotection by decreasing NO generation and enhancing neurite outgrowth [53]. The mechanism behind the increase in Th2 cells observed in the periphery and within the CNS following hAEC administration has not yet been elucidated. It has been shown that MSCs derived from adipose have the ability to upregulate chemokine receptor expression, such as CCR4, on peripheral lymphocytes [54]. It may be that hAECs are programming Th2 cells in the peripheral lymphoid organs to increase their expression of CCR4, which has been implicated in increased Th2 trafficking to the CNS during disease [55].

Although we did not use a control cell type in the current study, previous work, including our own, has clearly demonstrated that not all mouse and human cell types are capable of significantly attenuating clinical and pathological disease in EAE mice [28, 29, 56]. Based on this current literature, we believe that the beneficial effect observed in the current setting is due to the immunosuppressive and reparative properties of hAECs rather than a non-specific response to cell transplantation. Moreover, even though our results suggest that hAECs do not elicit a xenogeneic T cell response in vitro, we cannot rule out the possibility that the induction of an innate or humoral immune response to the injected cells may cause their subsequent rejection. Nevertheless, there is very strong evidence in current literature that shows long-term engraftment of transplanted cells is not required for therapeutic benefit [57–59].

## Conclusion

hAECs offer a new and promising alternative to other stem cell types that are easily obtained in the large quantities required for therapeutic transplantation. Our results demonstrate that hAECs posses potent immunomodulatory properties and protect mice from developing signs of RR-EAE when administered in the peritoneum. hAECs appear to elicit their immunomodulatory effects in EAE through promoting Tregs, maintaining the peripheral naïve CD4+ T cell pool and stimulating an anti-inflammatory Th2 environment, leading to the suppression of pathogenic T cell responses in peripheral secondary lymphoid organs and within the CNS. These attributes make hAECs an attractive cellular therapy for neurodegenerative diseases such as MS.

## References

[1] Ewing C, Bernard CCA (1998). Insights into the aetiology and pathogenesis of multiple sclerosis. Immunol Cell Biol..

[2] Lassmann H (2007). Multiple sclerosis: is there neurodegeneration independent from inflammation?. J Neurol Sci.

[3] Payne N, Siatskas C, Bernard CC (2008). The promise of stem cell and regenerative therapies for multiple sclerosis. J Autoimmun.

[4] Kieseier BC, Wiendl H, Leussink VI, Stuve O (2008). Immunomodulatory treatment strategies in multiple sclerosis. J Neurol..

[5] Li H, Niederkorn JY, Neelam S, Mayhew E, Word RA, McCulley JP (2005). Immunosuppressive factors secreted by human amniotic epithelial cells. Invest Ophthalmol Vis Sci.

[6] Whittle WL, Gibb W, Challis JR (2000). The characterization of human amnion epithelial and mesenchymal cells: the cellular expression, activity and glucocorticoid regulation of prostaglandin output. Placenta.

[7] Sakuragawa N, Kakinuma K, Kikuchi A, Okano H, Uchida S, Kamo I (2004). Human amnion mesenchyme cells express phenotypes of neuroglial progenitor cells. J Neurosci Res.

[8] Miki T, Mitamura K, Ross MA, Stolz DB, Strom SC (2007). Identification of stem cell marker-positive cells by immunofluorescence in term human amnion. J Reprod Immunol.

[9] Ilancheran S, Michalska A, Peh G, Wallace EM, Pera M, Manuelpillai U (2007). Stem cells derived from human fetal membranes display multilineage differentiation potential. Biol Reprod.

[10] Toda A, Okabe M, Yoshida T, Nikaido T (2007). The potential of amniotic membrane/amnion-derived cells for regeneration of various tissues. J Pharmacol Sci.

[11] Diaz-Prado S, Muinos-Lopez E, Hermida-Gomez T, Rendal-Vazquez ME, Fuentes-Boquete I, de Toro FJ (2010). Multilineage differentiation potential of cells isolated from the human amniotic membrane. J Cell Biochem.

[12] Banas RA, Trumpower C, Bentlejewski C, Marshall V, Sing G, Zeevi A (2008). Immunogenicity and immunomodulatory effects of amnion-derived multipotent progenitor cells. Hum Immunol.

[13] Murphy S, Rosli S, Acharya R, Mathias L, Lim R, Wallace E (2010). Amnion epithelial cell isolation and characterization for clinical use. Curr Protoc Stem Cell Biol.

[14] Miki T, Lehmann T, Cai H, Stolz DB, Strom SC (2005). Stem cell characteristics of amniotic epithelial cells. Stem Cells.

[15] Tan JL, Chan ST, Wallace EM, Lim R (2014). Human amnion epithelial cells mediate lung repair by directly modulating macrophage recruitment and polarization. Cell Transplant.

[16] Wolbank S, Peterbauer A, Fahrner M, Hennerbichler S, van Griensven M, Stadler G (2007). Dose-dependent immunomodulatory effect of human stem cells from amniotic membrane: a comparison with human mesenchymal stem cells from adipose tissue. Tissue Eng.

[17] McDonald C, Siatskas C, Bernard CCA (2011). The emergence of amnion epithelial stem cells for the treatment of multiple sclerosis. Inflamm Regen.

[18] Liu YH, Vaghjiani V, Tee JY, To K, Cui P, Oh DY (2012). Amniotic epithelial cells from the human placenta potently suppress a mouse model of multiple sclerosis. PLoS One.

[19] Moodley Y, Illancheran S, Samuel C, Vaghijani V, Atienza D, Williams ED (2010). Human amnion epithelial cell transplantation abrogates lung fibrosis and augments repair. Am J Respir Crit Care Med.

[20] Yawno T, Schuilwerve J, Moss TJ, Vosdoganes P, Westover AJ, Afandi E (2013). Human amnion epithelial cells reduce fetal brain injury in response to intrauterine inflammation. Dev Neurosci.

[21] Kudriashov AF, Artarian AA, Putsillo MV (1981). Use of amnion to repair a dural defect. Zh Vopr Neirokhir Im N N Burdenko..

[22] Mostaque AK, Abdur Rahman KB (2011). Comparisons of the effects of biological membrane (amnion) and silver sulfadiazine in the management of burn wounds in children. J Burn Care Res.

[23] Bujang-Safawi E, Halim AS, Khoo TL, Dorai AA (2010). Dried irradiated human amniotic membrane as a biological dressing for facial burns–a 7-year case series. Burns.

[24] Muraine M, Descargues G, Franck O, Villeroy F, Toubeau D, Menguy E (2001). Amniotic membrane graft in ocular surface disease. Prospective study with 31 cases. J Fr Ophtalmol.

[25] Zemba M, Andrei S, Cucu B, Bratulescu M, Stinghe A, Bobeico V (2006). Amniotic membrane transplantation in palliative treatment of bullous keratopathy. Oftalmologia.

[26] Motolese I, Mazzera L, Frezzotti P, Motolese PA, Motolese E (2010). Use of amniotic membrane transplantation in isolated conjunctival Bowen disease: a case report. Eur J Ophthalmol.

[27] Altan-Yaycioglu R, Akova YA, Oto S (2006). Amniotic membrane transplantation for treatment of symblepharon in a patient with recessive dystrophic epidermolysis bullosa. Cornea.

[28] Payne NL, Sun G, Herszfeld D, Tat-Goh PA, Verma PJ, Parkington HC (2012). Comparative study on the therapeutic potential of neurally differentiated stem cells in a mouse model of multiple sclerosis. PLoS One.

[29] Payne NL, Sun G, McDonald C, Layton D, Moussa L, Emerson-Webber A (2013). Distinct immunomodulatory and migratory mechanisms underpin the therapeutic potential of human mesenchymal stem cells in autoimmune demyelination. Cell Transplant.

[30] Short MA, Campanale N, Litwak S, Bernard CC (2011). Quantitative and phenotypic analysis of bone marrow-derived cells in the intact and inflamed central nervous system. Cell Adh Migr.

[31] Parolini O, Alviano F, Bagnara GP, Bilic G, Buuhring HJ, Evangelista M (2008). Concise review: isolation and characterization of cells from human term placenta: outcome of the first international Workshop on Placenta Derived Stem Cells. Stem Cells.

[32] Lim R, Chan ST, Tan JL, Mockler JC, Murphy SV, Wallace EM (2013). Preterm human amnion epithelial cells have limited reparative potential. Placenta.

[33] Dominici M, Le Blanc K, Mueller I, Slaper-Cortenbach I, Marini F, Krause D (2006). Minimal criteria for defining multipotent mesenchymal stromal cells. The International Society for Cellular Therapy position statement. Cytotherapy.

[34] Pratama G, Vaghjiani V, Tee JY, Liu YH, Chan J, Tan C (2011). Changes in culture expanded human amniotic epithelial cells: implications for potential therapeutic applications. PLoS One.

[35] Stadler G, Hennerbichler S, Lindenmair A, Peterbauer A, Hofer K, van Griensven M (2008). Phenotypic shift of human amniotic epithelial cells in culture is associated with reduced osteogenic differentiation in vitro. Cytotherapy.

[36] McQualter JL, Bernard CCA (2007). Multiple sclerosis: a battle between destruction and repair. J Neurochem.

[37] Hori J, Wang M, Kamiya K, Takahashi H, Sakuragawa N (2006). Immunological characteristics of amniotic epithelium. Cornea.

[38] Houlihan JM, Biro PA, Harper HM, Jenkinson HJ, Holmes CH (1995). The human amnion is a site of MHC class Ib expression: evidence for the expression of HLA-E and HLA-G. J Immunol.

[39] Kovats S, Main EK, Librach C, Stubblebine M, Fisher SJ, DeMars R (1990). A class I antigen, HLA-G, expressed in human trophoblasts. Science.

[40] Lovett-Racke AE, Trotter JL, Lauber J, Perrin PJ, June CH, Racke MK (1998). Decreased dependence of myelin basic protein-reactive T cells on CD28-mediated costimulation in multiple sclerosis patients. A marker of activated/memory T cells. J Clin Invest.

[41] Guan H, Nagarkatti PS, Nagarkatti M (2011). CD44 Reciprocally regulates the differentiation of encephalitogenic Th1/Th17 and Th2/regulatory T cells through epigenetic modulation involving DNA methylation of cytokine gene promoters, thereby controlling the development of experimental autoimmune encephalomyelitis. J Immunol.

[42] Brocke S, Piercy C, Steinman L, Weissman IL, Veromaa T (1999). Antibodies to CD44 and integrin alpha4, but not L-selectin, prevent central nervous system inflammation and experimental encephalomyelitis by blocking secondary leukocyte recruitment. Proc Natl Acad Sci U S A.

[43] Williams JL, Kithcart AP, Smith KM, Shawler T, Cox GM, Whitacre CC (2011). Memory cells specific for myelin oligodendrocyte glycoprotein (MOG) govern the transfer of experimental autoimmune encephalomyelitis. J Neuroimmunol.

[44] Slavin AJ, Johns TG, Orain JM, Bernard CC (1997). Regulation of myelin oligodendrocyte glycoprotein in different species throughout development. Dev Neurosci.

[45] Khoury SJ, Hancock WW, Weiner HL (1992). Oral tolerance to myelin basic protein and natural recovery from experimental autoimmune encephalomyelitis are associated with downregulation of inflammatory cytokines and differential upregulation of transforming growth factor beta, interleukin 4, and prostaglandin E expression in the brain. J Exp Med.

[46] Racke MK, Bonomo A, Scott DE, Cannella B, Levine A, Raine CS (1994). Cytokine-induced immune deviation as a therapy for inflammatory autoimmune disease. J Exp Med.

[47] Kohm AP, Carpentier PA, Anger HA, Miller SD (2002). Cutting edge: CD4 + CD25+ regulatory T cells suppress antigen-specific autoreactive immune responses and central nervous system inflammation during active experimental autoimmune encephalomyelitis. J Immunol.

[48] Tan JL, Chan ST, Lo CY, Deane JA, McDonald CA, Bernard CC (2015). Amnion cell mediated immune modulation following bleomycin challenge: controlling the regulatory T cell response. Stem Cell Res Ther.

[49] Pianta S, Bonassi-Signoroni P, Muradore I, Rodrigues MF, Rossi D, Silini A, et al. Amniotic membrane mesenchymal cells-derived factors skew T cell polarization toward Treg and downregulate Th1 and Th17 cells subsets. Stem Cell Rev. 2014; doi:10.1007/s12015-014-9558-4. 10.1007/s12015-014-9558-4 PMC4451472 25348066

[50] Parolini O, Souza-Moreira L, O’Valle F, Magatti M, Hernandez-Cortes P, Gonzalez-Rey E (2014). Therapeutic effect of human amniotic membrane-derived cells on experimental arthritis and other inflammatory disorders. Arthritis Rheumatol.

[51] Furlan R, Poliani PL, Galbiati F, Bergami A, Grimaldi LM, Comi G (1998). Central nervous system delivery of interleukin 4 by a nonreplicative herpes simplex type 1 viral vector ameliorates autoimmune demyelination. Hum Gene Ther.

[52] Butti E, Bergami A, Recchia A, Brambilla E, Del Carro U, Amadio S (2008). IL4 gene delivery to the CNS recruits regulatory T cells and induces clinical recovery in mouse models of multiple sclerosis. Gene Ther.

[53] Lisak RP, Nedelkoska L, Studzinski D, Bealmear B, Xu W, Benjamins JA (2011). Cytokines regulate neuronal gene expression: differential effects of Th1, Th2 and monocyte/macrophage cytokines. J Neuroimmunol.

[54] Razmkhah M, Jaberipour M, Erfani N, Habibagahi M, Talei AR, Ghaderi A (2011). Adipose derived stem cells (ASCs) isolated from breast cancer tissue express IL-4, IL-10 and TGF-beta1 and upregulate expression of regulatory molecules on T cells: do they protect breast cancer cells from the immune response?. Cell Immunol.

[55] Komiya T, Sugiyama T, Takeda K, Watanabe N, Imai M, Kokubo M (2013). Suppressive effects of a novel CC chemokine receptor 4 antagonist on Th2 cell trafficking in ligand- and antigen-induced mouse models. Eur J Pharmacol.

[56] Wang X, Kimbrel EA, Ijichi K, Paul D, Lazorchak AS, Chu J (2014). Human ESC-derived MSCs outperform bone marrow MSCs in the treatment of an EAE model of multiple sclerosis. Stem Cell Reports.

[57] Lee RH, Pulin AA, Seo MJ, Kota DJ, Ylostalo J, Larson BL (2009). Intravenous hMSCs improve myocardial infarction in mice because cells embolized in lung are activated to secrete the anti-inflammatory protein TSG-6. Cell Stem Cell.

[58] Mathias LJ, Khong SM, Spyroglou L, Payne NL, Siatskas C, Thorburn AN (2013). Alveolar macrophages are critical for the inhibition of allergic asthma by mesenchymal stromal cells. J Immunol.

[59] Chen L, Coleman R, Leang R, Tran H, Kopf A, Walsh CM (2014). Human neural precursor cells promote neurologic recovery in a viral model of multiple sclerosis. Stem Cell Reports.

